# A Lightweight YOLOv5-MNE Algorithm for SAR Ship Detection

**DOI:** 10.3390/s22187088

**Published:** 2022-09-19

**Authors:** Lei Pang, Baoxuan Li, Fengli Zhang, Xichen Meng, Lu Zhang

**Affiliations:** 1 School of Geomatics and Urban Spatial Informatics, Beijing University of Civil Engineering and Architecture, Beijing 102616, China; 2 Aerospace Information Research Institute, Chinese Academy of Sciences, Beijing 100094, China; 3 College of Geodesy and Geomatics, Shandong University of Science and Technology, Shandong 266000, China; 4 China Land Surveying and Planning Institute, Beijing 100032, China

**Keywords:** deep learning, YOLOv5, ship detection, lightweight model

## Abstract

Unlike optical satellites, synthetic aperture radar (SAR) satellites can operate all day and in all weather conditions, so they have a broad range of applications in the field of ocean monitoring. The ship targets’ contour information from SAR images is often unclear, and the background is complicated due to the influence of sea clutter and proximity to land, leading to the accuracy problem of ship monitoring. Compared with traditional methods, deep learning has powerful data processing ability and feature extraction ability, but its complex model and calculations lead to a certain degree of difficulty. To solve this problem, we propose a lightweight YOLOV5-MNE, which significantly improves the training speed and reduces the running memory and number of model parameters and maintains a certain accuracy on a lager dataset. By redesigning the MNEBlock module and using CBR standard convolution to reduce computation, we integrated the CA (coordinate attention) mechanism to ensure better detection performance. We achieved 94.7% precision, a 2.2 M model size, and a 0.91 M parameter quantity on the SSDD dataset.

## 1. Introduction

China has more than 18,000 km of coastline and a maritime land area of more than 300 square kilometers. Maritime security plays a vital role in our national defense security. However, in recent years, illegal fishing, drug trafficking, illegal immigration, and other illegal maritime activities are common, and some countries often send ships to perform “tours” in China’s waters. Therefore, ship detection technology plays an important role in protecting homeland security and monitoring illegal maritime activities [1].

Synthetic aperture radar (SAR) is an active earth observation system that can be installed on aircraft, satellites, spacecraft, and other flight platforms. It has a strong penetration ability to cloud, fog, rain, and so on, and is unaffected by light. It can observe the earth all day and in all weather conditions in real time and has a certain surface penetration ability [2]. Therefore, the SAR system has unique advantages in disaster, environmental, and marine monitoring [1,2,3,4,5]; resource exploration; crop yield estimation; surveying; mapping; military operations [4,5,6,7]; and other applications. It also plays a role that other remote sensing methods find difficult to play. Therefore, increasingly more countries have paid attention to it.

Owing to the characteristics of SAR imagery, ship detection using SAR images has become an important research direction for SAR image applications [1,2,3,4,5,6,7]. We can obtain several high-resolution SAR images through airborne and spaceborne SAR images. In these images, ships on the ocean are clearly seen. Therefore, we can use relevant images to detect ships and other targets conducive to improving the coastal defense capability of our country.

At present, there are many researches on deep learning [8,9,10,11]. Traditional ship target monitoring uses SAR images, and commonly used methods include template matching [12], support vector machine [13], linear interpolation [14], principal component analysis [15], a combination of multimode dictionary learning and sparse representation [16], and CFAR (constant false alarm rate) [17], and so forth. SAR images are easily affected by various environmental factors, such as speckle noise and background clutter, which makes it difficult to extract the features of the target of interest. The traditional method usually uses manual extraction, but there are some disadvantages, such as less feature extraction and difficult manual selection, and the rate of missing alarm is high, which ultimately affects the detection effect. Therefore, more and more scholars and scientific research institutions begin to carry out research on real-time detection of SAR ship targets.

Since the ImageNet competition in 2012, deep learning has begun to develop rapidly. Its powerful data processing and feature learning abilities have attracted people’s attention and recognition. Convolutional neural network (CNN) is commonly used in deep learning [9,10,11], more and more scholars have made a lot of research on this aspect [18,19,20,21,22,23,24,25,26,27,28,29,30]. However, most current algorithms [31,32,33,34,35] focus on improving the model’s accuracy, ignoring its speed and high cost. For some large companies, the pretraining large model optimizes the algorithm and improves accuracy, but for smaller companies, the expensive cost of training is prohibitive. In addition, the accuracy and speed of the model are not always perfect. Improved accuracy decreases model speed. In the case that much time is needed, it appears weak in instances that need real-time detection. Therefore, through lightweight models, network redundancy can be compressed and reduced, which greatly reduces the storage capacity, effectively improves the training speed and efficiency of the model [36,37], and orients it to real-time to achieve a broader range of real-time detection.

Different from traditional methods that require manual feature designs, deep learning methods can automatically extract features to achieve end-to-end target detection. Furthermore, the detection performance of deep-learning-based methods is superior. Generally speaking, target detection methods based on deep learning can be divided into two categories. The first is single-stage detection, and the mainstream single-stage detection models include the YOLO (You Only Look Once) series [1,2] and SSD (Single Shot MultiBox Detector) algorithm [38]. Based on regression, this method can directly predict the category confidence and locate the target position on the image. The other is the two-stage model, which presents the regional proposal network structure, generates a series of candidate boxes containing potential targets, and then further determines the target category and corrects the boundary boxes. Faster R-CNN [9], Feature Pyramid Networks (FPNs) [10], Mask R-CNN [11], and other algorithms based on multiscale feature fusion have been developed. The detection speed of the single-stage model is superior, and the effect of real-time detection is achieved. The detection accuracy of the two-stage model is better.

In terms of target detection, Dong et al. [31] improved the Faster RCNN by replacing the traditional nonmaximum suppression (NMS) with a Sig-NMS in the regional proposal network stage, significantly reducing the possibility of small missing targets. Cui et al. [32] proposed a detection method based on the intensive attention Pyramid Network (DAPN). Extracting rich features, including resolution and semantic information, improves the detection performance of multiscale ship targets. For multidirection target detection, An et al. [33] improved DRbox-V1 by FPN, focal loss, and improved coding scheme, and proposed the drbox-V2 detector, which detects ships in any direction. Li et al. [34] proposed a residual network based on a rotating region (R3-NET) to detect multidirectional vehicles for remote sensing images and videos with high robustness and accuracy. For dense target detection, Wang et al. [35] added the Spatial Group-wise Enhancement (SGE) attention module to CenterNet, which detected densely docked ships well. However, although the above methods have achieved satisfactory progress of detection accuracy, they are still computationally expensive, time-consuming, and unsuitable for deploying devices with limited computing resources and memory. Therefore, it is necessary to design a lightweight target detection model for remote sensing images.

In terms of target detection using SAR images, Feng et al. [1] proposed a new lightweight position-enhanced anchor-free SAR ship detection algorithm called LPEDet, which redesigned the lightweight multiscale backbone for a new position-enhanced attention strategy. Xu et al. [2] designed a lightweight cross stage part (L-CSP) module to reduce the amount of computation and applied network pruning for a more compact detector. The FASC-NET proposed by Yu et al. [26] is mainly composed of ASIR block, focus block, SPP block, and CAPE block; this network can reduce the number of parameters to a certain extent and maintain a certain accuracy without losing information. Then, to ensure excellent detection performance, Hou et al. [39] proposed a ship detection method for SAR images based on a visual attention model featuring the existing priors of ships in the water. This method can accurately detect ocean-going ships; however, berthing ships’ missed detection and false alarm rates are high. Liu and Cao [40] proposed a SAR image target detection method based on a visual attention pyramid model and singular value decomposition (VA-SVD), which has a slow calculation speed and poor detection performance for high-resolution SAR images. Wang et al. [41] proposed a target detection algorithm for high-resolution SAR images applied to complex scenes based on visual attention with high detection accuracy, but it cannot retain the original shape of the target. Yu et al. [42] proposed an efficient lightweight network, Efficient-YOLO. In this paper, a new regression loss function, ECIoU, is proposed to improve positioning accuracy and model convergence speed, the SCUPA module is proposed to enhance the generalization ability of the model, and the GCHE module is proposed to enhance the feature extraction ability of the network. Jiang et al. [43] proposed a three-channel image construction scheme based on NSLP contour extraction, which enriches the contour information of the dataset while reducing the impact of noise. Liu et al. [44] proposed a lightweight YOLOV4-Lite model based on which the MobileNetv2 network was used as the backbone feature extraction network, and deep separable convolution was used to reduce the computational overhead in the process of network training and ensure the lightweight characteristics of the network. Sun et al. [45] proposed a novel YOLO-based arbitrary-oriented SAR ship detector using bidirectional feature fusion and angular classification (BiFA-YOLO). This paper will be a novel bidirectional feature fusion module (BI-DFFM) specifically for SAR ship detection applied to the YOLO framework to effectively aggregate multiscale features to detect multiscale ships, and an angle classification structure is added to obtain ship angle information more accurately. 

At present, significant research has been conducted on SAR ship monitoring [1,2,3,4,5,6,7]. There is a great difference between SAR images and optical images. SAR images are generally used for target detection only with amplitude information. Meanwhile, SAR images are susceptible to various environmental factors, such as speckle noise and background clutter, which complicates feature extraction of interesting targets. In addition, the movement sensitivity and pose sensitivity of the sensor also lead to SAR target instability. The target detection algorithm of optical images is not entirely applicable to SAR images.

In summary, the following problems must be resolved:

(1) In order to further improve the accuracy of existing algorithms, most work involves blindly increasing the structure of the model, resulting in a large number of model parameters that slow down the speed of model training and reasoning. This outcome is not only not conducive to the real-time detection effect of the model, but also reduces the practicality of the model. At the same time, the complexity and the number of parameters of the model also limit the application and promotion of the model to a certain extent.

(2) Some models may not consider the problem of location information and computation overhead, which may lead to inaccurate target positioning during target detection, or the detection effect is not good because the hardware equipment with higher conditions is required.

To this end, we propose a new lightweight YOLOv5-MNE that improves the training speed and reduces the memory of SAR ship detection, and we did many ablation studies to compare. The main contributions are as follows:

(1) To solve the model speed reduction problem caused by a high number of parameters in the model, we designed a lightweight module, MNEBlock. Based on the YOLOv5 [46] network, a lightweight YOLOv5-MNE network was formed by fusing MNEBlock into the backbone of the basic network.

(2) In order to help the model locate and identify the objects of interest more accurately, the CA (coordinate attention) mechanism is introduced into this paper. The CA mechanism is flexible and lightweight, which avoids a lot of computational overhead and compensates the accuracy to a certain extent.

(3) Extensive ablation experiments were performed to confirm the validity of these contributions. The same experiment was performed on different datasets to compare the applicability of the proposed method with different datasets. Experiments were conducted on different orders of magnitude datasets to compare the applicability of the proposed method for different orders of magnitude datasets.

The remaining materials are arranged as follows: Section 2 describes the method used in this paper. Section 3 describes the results of these experiments. Section 4 describes the ablation experiments. Finally, Section 5 summarizes the whole article and presents our conclusions.

## 2. Methodology

This section describes the main ideas of YOLOV5-MNE in detail. Section 2.1 describes the network architecture of YOLOv5. Section 2.2 introduces the network architecture of YOLOV5-MNE, and Section 2.3 introduces the improved and added parts of this paper.

### 2.1. Network Structure of YOLOv5

YOLO series algorithms [1,2] are commonly used for their simple structure and fast processing speed. YOLOv5 is a single-stage target detection algorithm that adds some new and improved ideas based on YOLOv4. It is a relatively advanced target detection algorithm with fast inference speed and high accuracy. The main network structures are classified into four types, namely, YOLOv5s, YOLOv5m, YOLOv5l, and YOLOv5x. Considering the problem of deploying to devices with limited memory and computing resources, we chose YOLOv5s with a smaller size and faster speed after comprehensive consideration.

**Figure 1:**
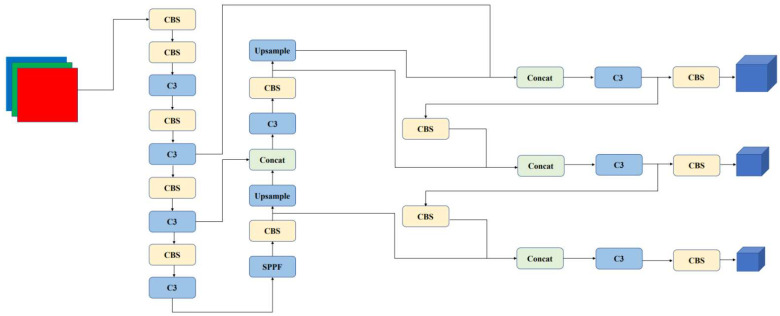
The framework of YOLOv5s. The network consists of five parts: input, backbone, neck, prediction, and output.

The YOLOv5 system consists of five parts: input, trunk, neck, prediction, and output. Figure 1 shows the network structure of YOLOv5. Compared with the previous version, the main differences in this version are as follows: (1) The focus layer is deleted; previously, the first layer of the network was the focus layer. It was changed to a convolution layer with kernel = 6, stride = 2, and padding = 2. Comparatively speaking, the modified convolution layer reduces the information loss caused by downsampling, which sacrifices a little accuracy to improve speed and achieves higher efficiency. (2) The SiLU activation function. SiLU is used for almost all activation functions in this version of the network architecture. (3) The SPPF module. In prior versions of YOLOv5, the neck adopted the SPP module. The SPP module uses the maximum pooling mode of kernels 1*1, 5*5, 9*9, and 13*13 for concat feature maps of different sizes. The SPPF structure serializes the input through multiple MaxPool layers of 5*5; that is, the 9*9 convolution is replaced by two 5*5 convolutions, and three 5*5 convolution operations replace the 13*13 convolution.

These modules constitute the target detection network. The prediction part of YOLOv5 consists of three layers of different scales: 20*20, 40*40, and 80*80. Among them, the small-scale detection head is suitable for targeting large ships, the medium-scale detection head is suitable for targeting medium-sized ships, and the large-scale detection head is suitable for targeting small ships.

### 2.2. Network Structure of YOLOv5—MNE

**Figure 2:**
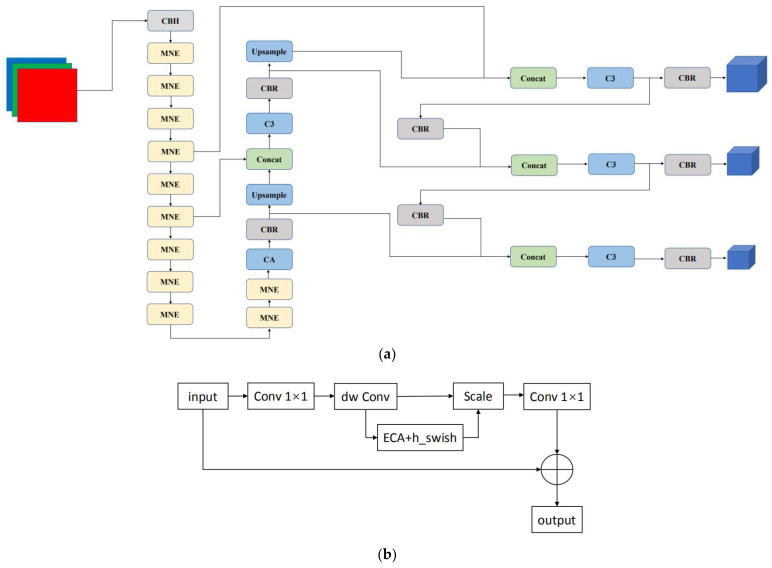
The framework of YOLOv5-MNE ((**a**) the framework of YOLOv5-MNE; (**b**) part of the framework of MNEBlock).

Although YOLOv5s is smaller and faster in the YOLOv5 series, it also requires a many parameters and a considerable amount of GPU memory. As shown in Figure 2, based on YOLOv5s, this paper designs a lighter MNEBlock to be integrated into the backbone of YOLOv5 and replaces the CBS convolution layer in the network with CBL standard convolution with fewer parameters. These operations reduce the number of parameters and memory consumption to a certain extent. Considering that the accuracy will be reduced, this paper adds a CA mechanism [8].

For the lightweight network design, we first added the ECA (efficient channel attention) attention module to MobileNetV3 [47] to form a MobileNet-ECA Block, significantly reducing network parameters and improving detection efficiency. To increase the smoothness of the subsequent compression, the ReLU function was adopted and substituted into the convolution layer, thus replacing the CBS (Conv_BN_SiLU) convolution layer with the CBR (Conv_BN_ReLU) convolution layer.

For better feature extraction and superior precision, we introduced the CA mechanism in the backbone [8]. The difference between the CA mechanism [8] and the traditional attention mechanism lies in its location information, which increases accuracy and efficiency in target positioning and detection.

In Section 2.3, we discuss details about the added modules.

### 2.3. Module of Design

#### 2.3.1. MNEBlock

MNEBlock is based on MobileNetV3. MobileNetV3 is a lightweight network architecture proposed by Google on 21 March 2019. Its network architecture is based on MnasNet implemented by NAS, which NAS searches to obtain parameters. The separable convolution mentioned in MobileNetV1 and the backward residual structure of the linear bottleneck mentioned in MobileNetV2 were introduced. MobileNetV3 introduces the lightweight attention model based on the SE (squeeze and excitation) structure [48] and uses a new activation function, h-swish(x). MNEBlock, based on MobileNetV3, eliminates the original SE attention mechanism and incorporates ECA. Thus, the model has fewer parameters, less memory, and higher accuracy. Table 1 shows the YOLOv5-MNE backbone network. 

**Figure 3:**
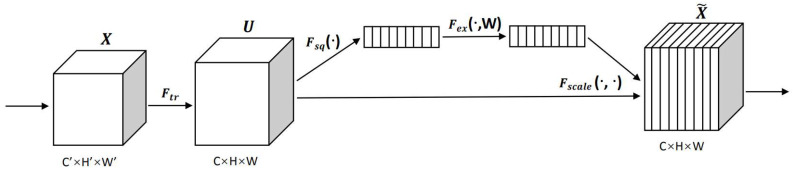
The network of SE.

The SE attention mechanism has been used in many networks. The SE attention mechanism enhances the channel-level feature response by modeling the interdependence between channels so that the important features can be strengthened and the nonimportant features weakened. The model can obtain better features by weighting the channels in the network. The network of SE is shown in Figure 3. 

The SE attention mechanism connects CAM (channel attention module) and SAM (spatial attention module), with one focusing on “what” and the other on “where”. The steps are as follows:

(a) First, given the input *X*, the squeeze step for the *c*-th channel can be formulated as:

(1) $$z_{c}=\frac{1}{H\times W}\sum_{i=1}^{H}\sum_{j=1}^{W}x_{c}\left(i,\;j\right)$$

where $z_{c}$ is the output associated with the *c*-th channel. The input X is directly from a convolutional layer with a fixed kernel size and, hence, can be viewed as a collection of local descriptors.

(b) The squeeze operation makes collecting global information possible,

(2) $$\hat{X}=X\cdot \sigma \left(\hat{z}\right)\;$$

where X refers to channel-wise multiplication; σ is the sigmoid function; and $\hat{z}$ is the result generated by a transformation function, which is formulated as follows:

(3) $$\hat{z}=T_{2}\left(ReLU\left(T_{1}\left(z\right)\right)\right)\;$$

where $T_{1}$ and $T_{2}$ are two linear transformations that capture the importance of each channel.

**Figure 4:**
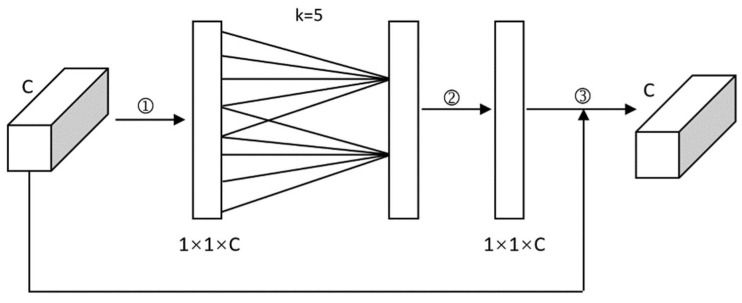
The network of ECA (①: given the aggregated features obtained by global average pooling (GAP); ②: σ; ③: ×).

The SE attention mechanism carries on the channel compression to the characteristics of the input figure. However, such compression dimension reduction does not benefit studying the channel dependency relationship. Therefore, the ECA mechanism [49], to avoid dimension reduction with one-dimensional convolution, must efficiently implement a local cross-modal interaction that is more conducive to extracting the dependent relationships between channels. The network of ECA is shown in Figure 4, and the steps are as follows:

(a) Global average pooling of the input feature maps;

(b) One-dimensional convolution operation with convolution kernel size *k* is conducted. The weight w of each channel is obtained through the sigmoid activation function,

(4) $$w=\sigma \left(C1D_{k}\left(y\right)\right)\;$$

where *σ* is the sigmoid function.

(c) The weights are multiplied by the corresponding elements of the original input feature map to obtain the final output feature map.

#### 2.3.2. CA Mechanism

**Figure 5:**
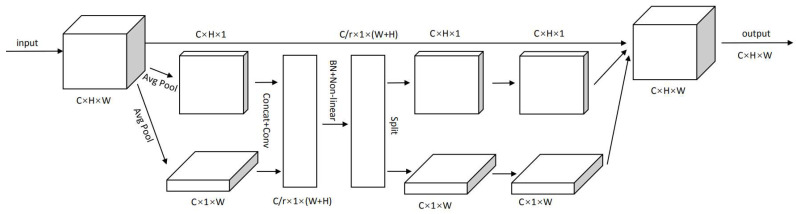
The network of CA.

Existing attention mechanisms generally use maximum or average pooling to manage channels, which lose spatial information to a certain extent. In lightweight networks where model capacity is strictly limited, the computing overhead of attention application is even more unaffordable. However, the CA mechanism [8] embeds location information in channel attention, making mobile networks participate in a larger area to avoid a large amount of computing overhead. The network of CA is shown in Figure 5. 

The CA mechanism [8] divides channel attention into two features encoding along different directions. These features preserve precise location information about one spatial direction while capturing the dependencies of the other. Both are complementary to the input feature graph to enhance the representation of the object of interest. The steps are as follows:

(a) The input feature maps underwent global average pooling along the width and height directions to obtain feature maps for both directions:

(5) $$z_{c}^{h}\left(h\right)=\frac{1}{W}\sum_{0\leq i<W}x_{c}\left(h,\;i\right)$$

(6) $$z_{c}^{w}\left(w\right)=\frac{1}{H}\sum_{0\leq j<H}x_{c}\left(j,w\right)$$

(b) The feature graphs of two spatial directions are concatenated and normalized by convolution,

(7) $$f=\delta \left(F_{1}\left(\left[z^{h},z^{w}\right]\right)\right)$$

where δ is the nonlinear activation function and $f=R^{\frac{C}{r}*(H+W)}$ is the intermediate feature map that encodes spatial information in both the horizontal and vertical directions.

(c) It decomposes into convolution of height and width along the dimension of space, and is activated by the sigmoid function,

(8) $$g^{h}=\sigma \left(F_{h}\left(f^{h}\right)\right)$$

(9) $$g^{w}=\sigma \left(F_{w}\left(f^{w}\right)\right)$$

(10) $$y_{c}\left(i,j\right)=x_{c}\left(i,j\right)\times g_{c}^{h}\left(i\right)\times g_{c}^{w}\left(j\right)$$

where *σ* is the sigmoid function.

#### 2.3.3. CBR (Conv-BN-ReLU)

In a neural network, there is a functional relationship between the output of the upper node and the input of the lower node called the activation function. The activation function introduces nonlinear factors to the neural network and can be used to fit various curves, satisfying the required relationship between the output of the upper node and the input of the lower node.

**Figure 6:**
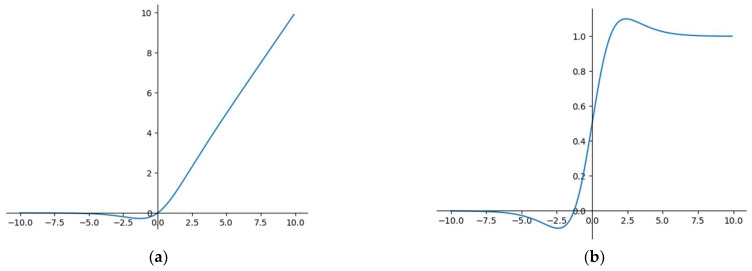
The graph of SiLU: (**a**) is the SiLU function graph; (**b**) is the derivative SiLU function graph.

The convolution in YOLOv5s uses the SiLU activation function, which has no upper or lower bounds. It can avoid overfitting to some extent and produce stronger regularization effects. The SiLU function is of great distribution significance and is a function of both the swish and ReLU functions. Figure 6 shows the graph of SiLU. The formula for SiLU is as follows:

(11) $$SiLU\left(x\right)=x*\mathrm{Sigmoid}\left(x\right)$$

**Figure 7:**
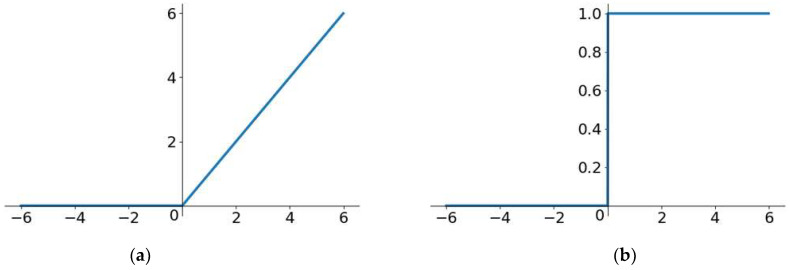
The graph of ReLU: (**a**) is the graph of the ReLU function; (**b**) is the derivative graph of the ReLU function.

We changed the activation function of the convolution layer into the commonly used ReLU function, which solved the problem of gradient disappearance. Moreover, due to the linear and unsaturated forms of the ReLU function, it converges rapidly into SGD. Most importantly, the ReLU activation function only has linear relations and does not need exponential calculation; therefore, its operation speed is much faster in both forward and backward propagation. Therefore, we used the CBR standard convolution, including the ReLU activation function, in this paper to improve the speed of the lightweight network. Figure 7 shows the graph of ReLU. The formula for ReLU is as follows:

(12) $$f\left(x\right)=\max\left(0,\;x\right)$$

## 3. Experiments

To verify the proposed method, we conducted a series of related experiments to evaluate the model’s detection performance. The content of this section includes details of some settings in the experiment and the main content of the SSDD dataset, followed by the evaluation indicators used in the experimental results.

### 3.1. Experimental Platform

We used a workstation with the GPU model of NVIDIA RTX3080, CPU model of i9-11900K, and memory size of 32 G to conduct the training part of the experiment. PyTorch 1.9.1, based on the Python 3.8 language, was adopted as the framework of our algorithm. We also used CUDA 11.1 in our experiments to call the GPU for training acceleration.

### 3.2. Dataset

In our experiments, we used SSDD [50] and AIR-SARship 1.0 [51] for training and validation. For each ship, the detection algorithm predicts the frame of the ship target and its confidence. To make a fair comparison with another work, we randomly divided the original dataset according to the ratio of 8:2 commonly used in existing studies. Of that, 80% of the dataset was used for the training of all methods, and the remaining 20% was used as a test set to evaluate the detection performance of all methods. In addition, considering the generalization ability of the model, we introduced a new dataset, HRSID dataset [52]; randomly selected part of the HRSID data as the test set; and tested the model trained for the SSDD dataset using the HRSID test set.

**Figure 8:**
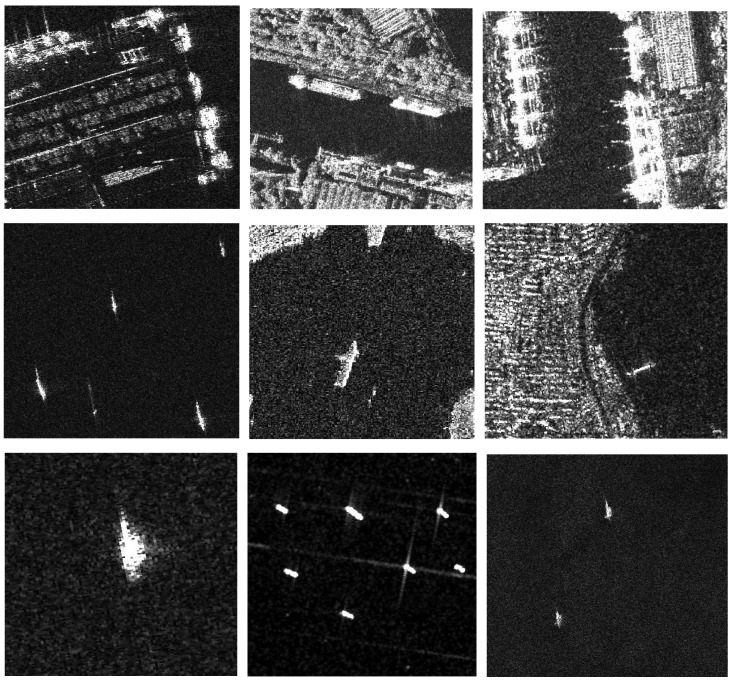
The ship target images in the SSDD dataset.

The SSDD dataset is widely used for SAR ship detection. In this article, SSDD data are obtained by downloading public SAR images from the internet. The data mainly include RadarSAT-2, TerraSAR-X, and Sentinel-1 sensors with four polarization modes, HH, HV, VV, and VH, with a resolution of 1m–15 m and ship targets in a large area of sea and near shore. Figure 8 shows part of the images in the dataset. The target area was cropped to approximately 500 × 500 pixels, and the ships’ target location was manually marked. There are 1160 images in the dataset, and each image contains ships of different numbers and sizes. Table 2 shows the statistical information of the average number of ships per image in the SSDD dataset.

**Figure 9:**
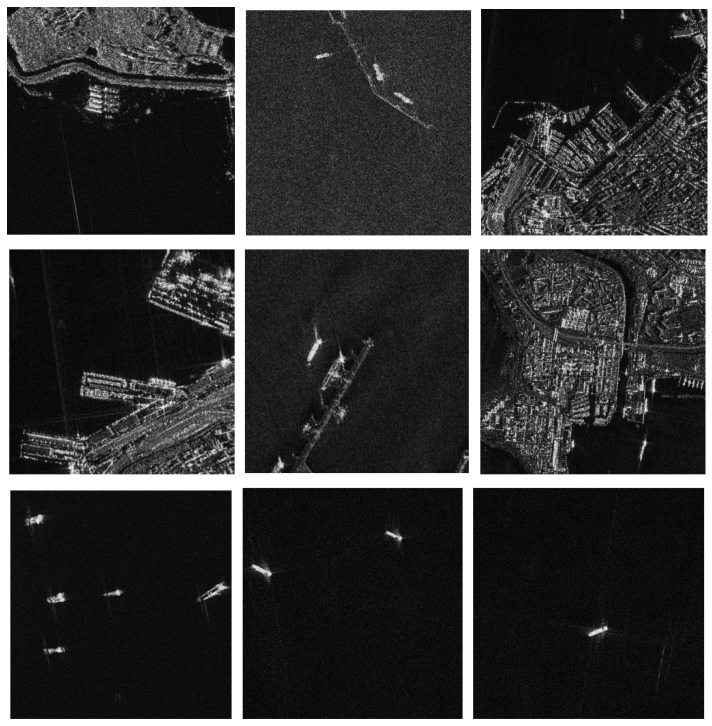
The ship target images in the AIR-SARship 1.0 dataset.

The AIR-SARship 1.0 dataset released 31 scenes of Gaofen-3 SAR images in the first batch, with image resolutions of 1 and 3 m. The targets cover nearly 1000 ships in more than 10 categories, such as transport ships, oil tankers, fishing boats, and so on. Scene types include ports, reefs, and sea surfaces of different sea states. Figure 9 shows part of the images in the dataset. Since no detailed distinction is made in the dataset, all ships in the dataset are defined as one class of this paper. In this paper, we cropped these images to approximately 500 × 500 pixels, with the overlapping part half the size of the image. We cropped a total of 651 SAR images containing ships.

**Figure 10:**
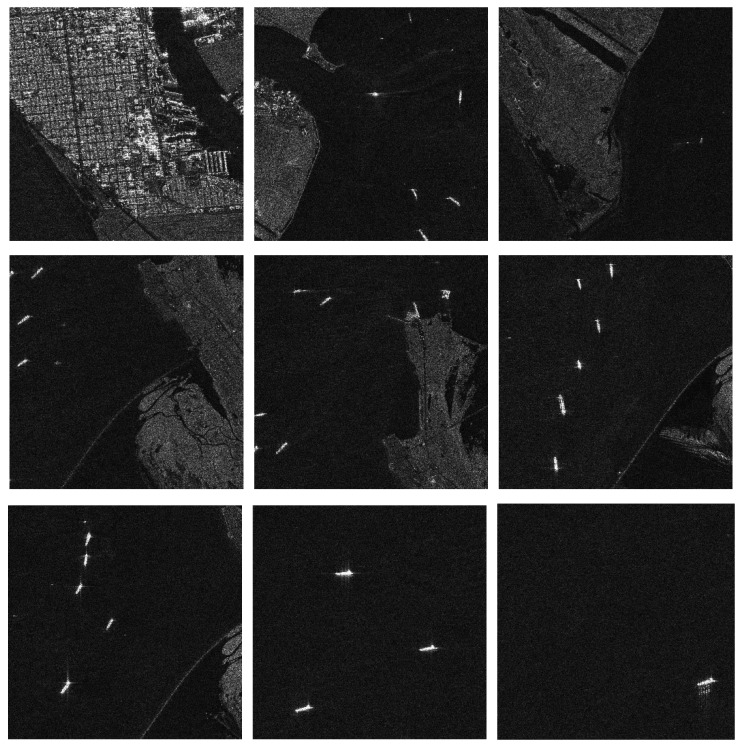
The ship target images in the HRSID dataset.

The HRSID dataset is a large-scale SAR target detection dataset. The images of the HRSID dataset are high-resolution SAR images from Sentinel-1B, TerraSAR-X, and Tandemi-X sensors, which are mainly used for ship detection, semantic segmentation, and instance segmentation tasks. The dataset contains a total of 5604 high-resolution SAR images and 16,951 ship instances. The resolutions of the HRSID contain 0.5, 1, and 3 m. Figure 10 shows part of the images of the dataset.

### 3.3. Experimental Details

We used the stochastic gradient descent (SGD) [53] algorithm to train our network. The network adopted the batch size of 16. We trained the network for 100 total epochs when we performed the ablation experiments. We also set the learning rate as 0.01, the weight decay as 0.0005, and the SGD momentum as 0.937. Other unmentioned hyper-parameters were kept the same as those in YOLOv5. In addition, when we compared with other methods, we set basically the same parameters in order to ensure a fair comparison.

### 3.4. Evaluation Indicators

We used average precision, recall, average precision (*mAP*), model size, parameter amount, and so on to analyze and verify the detection performance of our proposed method [2]. Average precision (*mAP*) can be derived from accuracy and recall.

Accuracy is the percentage of correctly identified targets in the test set. The percentage is defined by true positives (*TP*) and false positives (*FP*):

(13) $$P=\frac{TP}{TP+FP}$$

where *TP* means that the prediction of the classifier is positive, and the prediction is correct. *FP* indicates that the prediction of the classifier is positive, and the prediction is incorrect.

The recall rate is the probability that all positive samples in the test set are correctly identified, which derives from true positives (*TP*) and false negatives (*FN*):

(14) $$R=\frac{TP}{TP+FN}$$

where *FN* indicates that the prediction of the classifier is negative, and the prediction is incorrect.

Average accuracy (*mAP*) is based on the accuracy and recall rate. The graphical meaning is clearly seen in the coordinate axis, that is, the area under the accuracy and recall rate curve, which is defined as follows:

(15) $$mAP=\int_{0}^{1}P\left(R\right)dR$$

Finally, FPS is how many frames per second the network can detect, model volume denotes the size of the weight, params denotes the parameter amount, and gpu-mem denotes the GPU memory when training.

## 4. Experimental Results and Ablation Study

### 4.1. Experimental Results

#### 4.1.1. Experiments Results on SSDD Dataset

**Figure 11:**
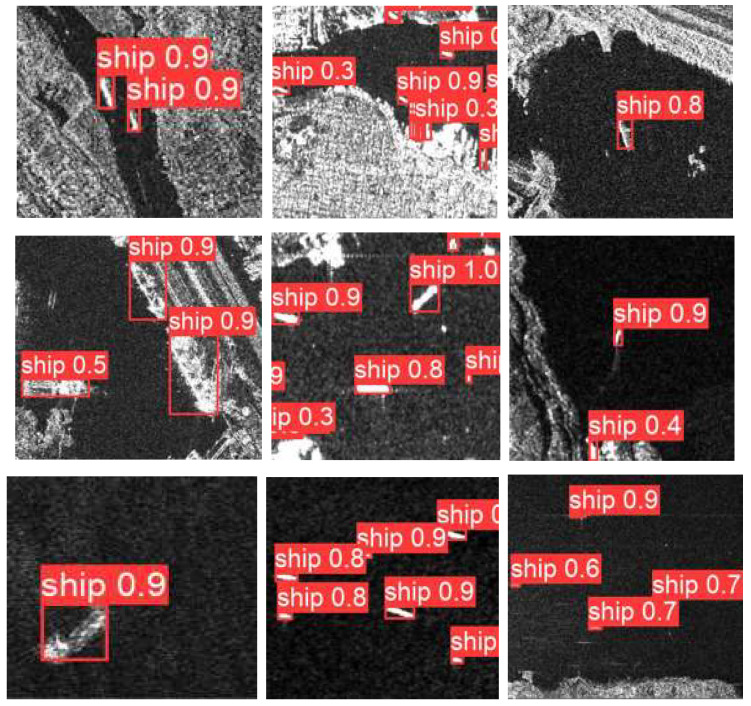
Visualization effect of our experiment on the SSDD dataset.

Our improved method based on YOLOv5. Figure 11 shows the visualization results on SSDD as an example. As we can see, our ship detection method has a good performance both inshore and offshore. The comparison between our method and YOLOv5 on SSDD dataset is shown in Table 3. Table 3 shows that although the precision of our method is reduced by 1.73% compared with YOLOv5, the model volume is reduced from 14.2 to 2.2 M, the params is reduced to 13%, and gpu-mem is reduced from 4.98 to 3.62 G.

To prove the validity of our method, we compared it with other methods used on the SSDD dataset, as shown in Table 4. Table 5 shows the comparison between our method and some lightweight methods on the SSDD dataset. In order to make the comparison fair, we set the parameters to be the same as those of these methods.

**Table 4:** Comparison between SAR ship detection methods on the SSDD dataset.

Methods	P (%)	R (%)	FPS	mAP (%)	Training Time (h)
**SL-CFAR** [54]	82.65	84.57	17.24	78.36	4.71
**NF** [55]	83.79	85.73	19.58	80.21	5.83
**CSEPD** [56]	81.24	82.76	21.26	77.43	6.52
**FBR-Net** [57]	86.73	87.14	38.52	83.76	5.42
**DPA-Net** [58]	87.56	88.26	41.86	84.25	4.83
**SSGE-Net** [59]	88.12	88.97	52.13	85.63	6.14
**ARR-Net** [60]	89.36	89.73	47.51	88.42	5.73
**Ours**	94.77	82.72	111.11	91.7	0.553

**Table 5:** Comparison between lightweight detection methods on the SSDD dataset.

Methods	mAP (%)	FPS	Model Volume (M)	Params (M)	GPU-MEM (G)
**BiFA-YOLO** [45]	94.85	75.19	39.4	19.57	-
**YOLOv4-LITE-MR** [44]	95.03	47.16	49.34	-	-
**YOLOv4-tiny** [43]	88.08	81.63	22.5	-	-
**Efficient-YOLO** [42]	93.56	66.14	31.34	8.2	-
**YOLO-v5-Light** [61]	88.7	-	23.9	3.01	-
**LPEDet** [1]	97.4	-	-	5.68	-
**Ours**	96.0	111.11	2.2	0.91	3.62

It can be seen from Table 4 and Table 5 that our method has certain advantages when compared with other ship detection methods. Our method not only has higher precision, but also has smaller parameter number and model volume compared with the other methods.

#### 4.1.2. Experimental Results on the AIR-SARship 1.0 Dataset

**Figure 12:**
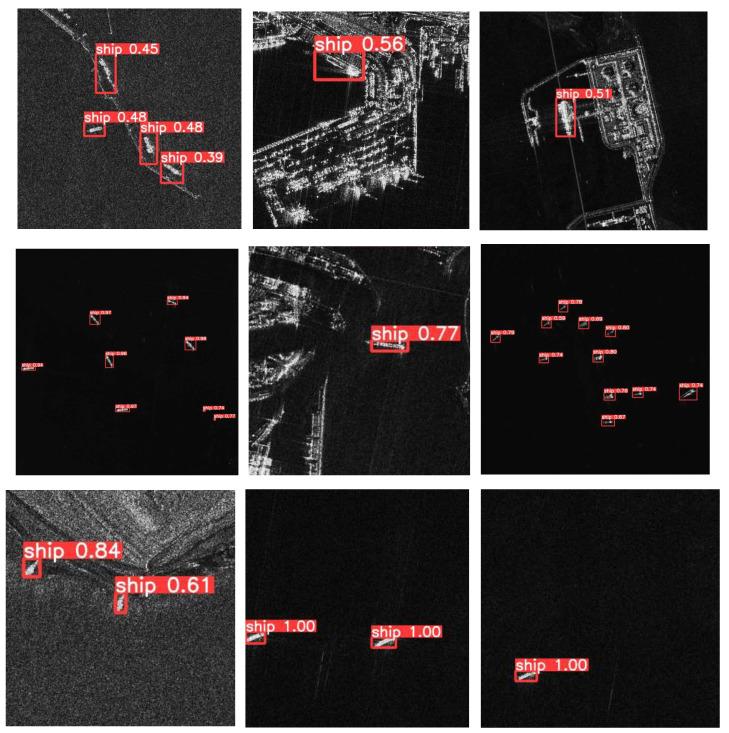
Visualization effect of our experiment on the AIR-SARship 1.0 dataset.

Our improved method based on YOLOv5. Figure 12 shows the visualization results on AIR-SARship 1.0 as an example. As we can see, our ship detection method has a better performance both inshore and offshore. The comparison between our method and YOLOv5 on the AIR-SARship 1.0 dataset is shown in Table 6. Table 6 shows that the precision of our method is reduced by 6.7% compared with YOLOv5, the model volume is reduced from 14.2 to 2.2 M, the params is reduced to 13%, and gpu-mem is reduced from 4.24 to 2.82 G.

To prove the validity of our method, we compared it with other methods used on the AIR-SARship 1.0 dataset, as shown in Table 7. As can be seen from Table 7, compared with the other methods, our method has better precision and training speed, but the recall and mAP are not ideal.

#### 4.1.3. Experimental Results on Different Datasets

**Figure 13:**
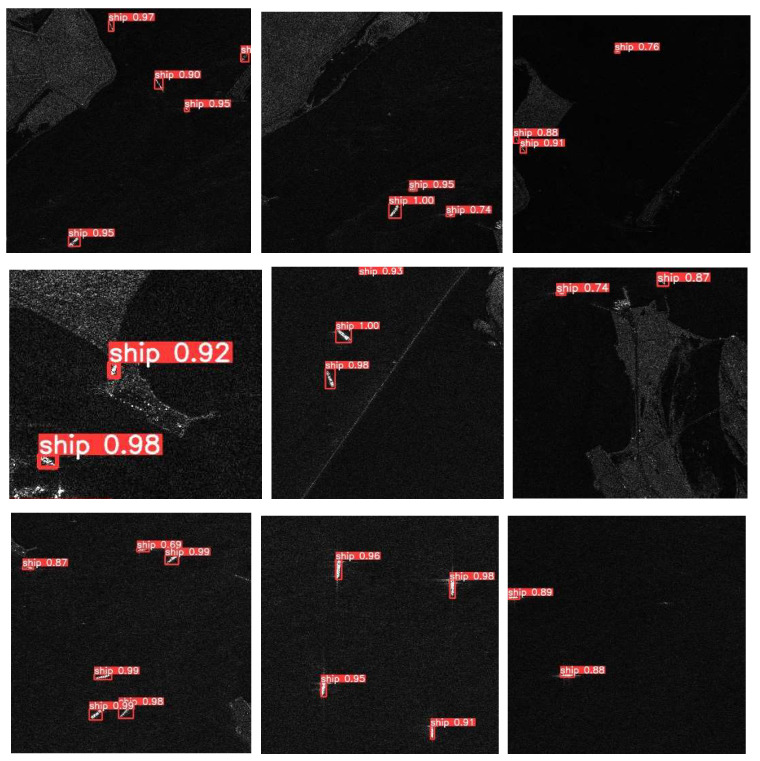
Visualization effect of our experiment on the HRSID dataset under the SSDD dataset training.

Considering the effect of train and test with different datasets, we used the SSDD dataset to train the model and the HRSID dataset as the test set to test the visualization results in this section. Figure 13 below shows the visualization results for testing the HRSID dataset on the basis of training on the SSDD dataset. As can be seen from Figure 13, when different datasets are used for the training set and testing set, our method has a better performance in detecting the ship targets both inshore and offshore.

### 4.2. Ablation Study

#### 4.2.1. Ablation Study on Different Modules

(1) Table 8 shows the ablation study of YOLOv5-MNE with different modules. We compared the original MobileNetV3 module with our MNEBlock. As can be seen from Table 8, our method improves the precision by 2.6% compared with the original MobileNetV3, the model volume is reduced from 3.2 to 2.3 M, and the params is reduced from 1.37 to 0.92 M.

(2) Table 9 shows the ablation study of YOLOv5-MNE with different modules. Here, we present three cases: (a) MobileNetV3 removes layer SE, (b) MobileNetV3, and (c) MNEBlock. Based on (a)–(c), we added different attention mechanisms to compare the effects. As can be seen from Table 9, the precision, after adding the CA mechanism; model volume; and params are slightly improved.

(3) Table 10 shows the ablation study of YOLOv5-MNE with different modules. Based on (2), we used different activations to compare the effects. As can be seen from Table 10, after replacing with the ReLU activation function, the precision, model volume, and params are also slightly improved.

(4) Table 11 shows the ablation study of YOLOv5-MNE with different modules. Based on (3), we used a different IoU to compare the effects. We find that using another IoU actually reduced our precision.

We compared each YOLOv5-MNE module to verify their effectiveness, where 1 represents the addition of only the MNEBlock module, 2 represents the addition of the MNEBlock module and CA mechanism, and 3 represents the addition of the MNEBlock module and CA mechanism while using the CBL standard convolution, which is the method in this paper. Among them, Table 12 shows the ablation study of our method on the SSDD dataset, and Table 13 shows the ablation study of our method on the AIR-SARship 1.0 dataset. We found that the addition of each step made our method better, and the accuracy gap between our method and YOLOv5s gradually narrowed.

#### 4.2.2. The Effect of the Number of SSDD Dataset on Detection Performance

To test the effect of our data at different quantities, we randomly selected 30% and 60% of the data from the SSDD dataset to validate the performance of our model. The results are shown in Table 14 below. The percentages of 30%, 60%, and 100% refer to what percentage of the original SSDD data we will use for training and testing. That is, we have 1160 SAR images when the dataset ratio is 100, which means that we will use 80% of all data, namely, 928 SAR images, for training, and the remaining 232 SAR images for testing. We have 696 SAR images when the dataset ratio is 60, which means that we will use 80% of 696 SAR images, namely, 557 SAR images, for training. The same goes for 30%. As we can see from Table 14, the precision of our method is affected as the total amount of data decreases. Comparing Table 12, Table 13 and Table 14, we consider that the accuracy of AIR-SARship 1.0 may also be affected by its small data volumes.

## 5. Conclusions

SAR ship detection has great application values in both the military and civil fields. We proposed a new algorithm, YOLOv5-MNE, to solve the problems of unclear contour information, complex backgrounds, and large models in SAR ship detection. (1) Design a lightweight module MNEBlock to solve the model speed reduction problem caused by a high number of parameters in the model. (2) Increase the CA mechanism to better use location information to improve accuracy. (3) Use the ReLU activation function to make the model more lightweight. After these improvements in YOLOv5, on the SSDD dataset, the precision of our method can reach 94.8%, the model volume can be reduced to 2.2 M, our gpu-mem is also reduced to 3.62 G, and at the same time, it can reach 111.11 FPS. However, on the AIR-SARship 1.0 dataset, although the number of parameters is also reduced, the accuracy is decreased by 6.7%. Therefore, we found that, in the case of sufficient data, although our algorithm has a slight loss of accuracy, it effectively reduces the running memory and model size of the model. However, when the data are insufficient, the effect will decrease. According to our research and ablation experiment results, our method is more suitable for lager datasets, while smaller datasets lead to a certain accuracy reduction. In the future, our work will be as follows: (1) We plan to lighten the detector further without sacrificing accuracy. (2) We plan to explore other approaches, such as knowledge distillation, quantification, and network pruning. (3) We will study how to achieve higher accuracy and better results with a small amount of training data.

## Figures and Tables

**Table 1:** Details on the YOLOv5-MNE backbone network.

Operator	Kernel Size	Stride	ECA	h-swish
**CBH**	3 × 3	1	-	√
**MNEBlock**	3 × 3	2	√	-
**MNEBlock**	3 × 3	2	-	-
**MNEBlock**	3 × 3	1	-	-
**MNEBlock**	5 × 5	2	√	√
**MNEBlock**	5 × 5	1	√	√
**MNEBlock**	5 × 5	1	√	√
**MNEBlock**	5 × 5	1	√	√
**MNEBlock**	5 × 5	1	√	√
**MNEBlock**	5 × 5	2	√	√
**MNEBlock**	5 × 5	1	√	√
**MNEBlock**	5 × 5	1	√	√
**CA**	-	-	-	-

**Table 2:** Correspondence between NS (number of ships) and NI (number of images) in the SSDD dataset.

NS	1	2	3	4	5	6	7	8	9	10	11	12	13
**NI**	725	183	89	47	45	16	1	8	4	11	5	3	3

**Table 3:** Performance comparison with the raw YOLOv5 on the SSDD dataset.

Methods	P (%)	Model Volume (M)	Params (M)	GPU-MEM (G)
**YOLOv5**	96.5	14.5	7.01	4.98
**YOLOv5-MNE** **(** **ours)**	94.77	2.2	0.91	3.62

**Table 6:** Performance comparison with the raw YOLOv5 on the AIR-SARship 1.0 dataset.

Methods	P (%)	Model Volume (M)	Params (M)	GPU-MEM (G)
**YOLOv5**	94.5	14.5	7.01	4.24
**YOLOv5-MNE (ours)**	87.8	2.2	0.91	2.82

**Table 7:** Comparison between SAR ship detection methods on the AIR-SARship 1.0 dataset.

Methods	P (%)	R (%)	FPS	mAP (%)	Training Time (h)
**SL-CFAR** [54]	82.58	84.65	16.58	78.32	9.53
**NF** [55]	83.75	85.73	18.73	79.54	8.24
**CSEPD** [56]	81.37	83.42	20.15	76.85	7.43
**FBR-Net** [57]	85.14	86.76	37.43	83.42	6.85
**DPA-Net** [58]	86.95	87.45	39.85	84.96	5.63
**SSGE-Net** [59]	87.56	88.93	50.23	86.73	7.14
**ARR-Net** [60]	88.92	89.58	45.24	87.25	6.58
**Ours**	87.8	70.6	116.28	78.5	0.719

**Table 8:** The ablation study of MNEBlock on the SSDD dataset.

Baseline	Methods	P (%)	Model Volume (M)	Params (M)
**YOLOv5**	MobileNetV3	90.3	3.2	1.37
	MNEBlock (ours)	92.9	2.3	0.92

**Table 9:** The ablation study of CA on the SSDD dataset.

Baseline	Methods	P (%)	Model Volume (M)	Params (M)
**a**	CA	92.6	2.2	0.91
**a**	ECA	92.9	2.2	0.91
**a**	PEA	92.6	2.2	0.91
**b**	CA	93.4	3.2	1.36
**c**	PEA	92.8	2.2	0.91
**Ours**	ours	93.2	2.2	0.91

**Table 10:** The ablation study of ReLU on the SSDD dataset.

Baseline	Methods	P (%)	Model Volume (M)	Params (M)
**(2)**	Aconc	89.8	2.3	0.91
	MetaAconc	90.6	3.4	0.98
	FReLU	92.3	2.3	0.93
	LeakyReLU	94.6	2.2	0.91
	DYReLUB	91.1	2.9	1.22
	Ours	94.8	2.2	0.91

**Table 11:** The ablation study of IoU on the SSDD dataset.

Baseline	Methods	P (%)	Model Volume (M)	Params (M)
**(2)+LeakyReLU**	AlpahIoU	90.2	2.2	0.91
**(2)+LeakyReLU**	EIoU	90.9	2.2	0.91
**(2)+ ReLU**	AlpahIoU	92.2	2.2	0.91
**(2)+ ReLU**	EIoU	90.8	2.2	0.91
**(2)+ ReLU**	SIoU	94.1	2.2	0.91
**(2)+ ReLU**	ours	94.8	2.2	0.91

**Table 12:** The ablation study on the SSDD dataset.

Methods	P (%)	Model Volume (M)	Params (M)	GPU-MEM (G)
**1**	92.9	2.3	0.92	3.7
**2**	93.2	2.2	0.91	3.72
**3 (ours)**	94.8	2.2	0.91	3.62

**Table 13:** The ablation study on the AIR-SARship 1.0 dataset.

Methods	P (%)	Model Volume (M)	Params (M)	GPU-MEM
**1**	79.1	2.3	0.92	2.88
**2**	87.6	2.2	0.91	2.89
**3 (ours)**	87.8	2.2	0.91	2.82

**Table 14:** Comparison of experimental results for different SSDD data volumes.

Methods	Dataset Ratio (%)	R (%)
**YOLOv5**	30	91.5
	60	97.8
	100	96.5
**YOLOv5-MNE (ours)**	30	87
	60	94.5
	100	94.77

## Data Availability

Not applicable.
